# Advances in the Application of Single-Cell Transcriptomics in Plant Systems and Synthetic Biology

**DOI:** 10.34133/bdr.0029

**Published:** 2024-02-29

**Authors:** Md Torikul Islam, Yang Liu, Md Mahmudul Hassan, Paul E. Abraham, Jean Merlet, Alice Townsend, Daniel Jacobson, C. Robin Buell, Gerald A. Tuskan, Xiaohan Yang

**Affiliations:** ^1^ Biosciences Division, Oak Ridge National Laboratory, Oak Ridge, TN 37831, USA.; ^2^ The Center for Bioenergy Innovation, Oak Ridge National Laboratory, Oak Ridge, TN 37831, USA.; ^3^ Department of Genetics and Plant Breeding, Patuakhali Science and Technology University, Dumki, Patuakhali 8602, Bangladesh.; ^4^ Bredesen Center for Interdisciplinary Research and Graduate Education, University of Tennessee Knoxville, Knoxville, TN 37996, USA.; ^5^ Center for Applied Genetic Technologies, University of Georgia, Athens, GA 30602, USA.; ^6^ Department of Crop and Soil Sciences, University of Georgia, Athens, GA 30602, USA.; ^7^ Institute of Plant Breeding, Genetics, and Genomics, University of Georgia, Athens, GA 30602, USA.

## Abstract

Plants are complex systems hierarchically organized and composed of various cell types. To understand the molecular underpinnings of complex plant systems, single-cell RNA sequencing (scRNA-seq) has emerged as a powerful tool for revealing high resolution of gene expression patterns at the cellular level and investigating the cell-type heterogeneity. Furthermore, scRNA-seq analysis of plant biosystems has great potential for generating new knowledge to inform plant biosystems design and synthetic biology, which aims to modify plants genetically/epigenetically through genome editing, engineering, or re-writing based on rational design for increasing crop yield and quality, promoting the bioeconomy and enhancing environmental sustainability. In particular, data from scRNA-seq studies can be utilized to facilitate the development of high-precision Build–Design–Test–Learn capabilities for maximizing the targeted performance of engineered plant biosystems while minimizing unintended side effects. To date, scRNA-seq has been demonstrated in a limited number of plant species, including model plants (e.g., *Arabidopsis thaliana*), agricultural crops (e.g., *Oryza sativa*), and bioenergy crops (e.g., *Populus* spp.). It is expected that future technical advancements will reduce the cost of scRNA-seq and consequently accelerate the application of this emerging technology in plants. In this review, we summarize current technical advancements in plant scRNA-seq, including sample preparation, sequencing, and data analysis, to provide guidance on how to choose the appropriate scRNA-seq methods for different types of plant samples. We then highlight various applications of scRNA-seq in both plant systems biology and plant synthetic biology research. Finally, we discuss the challenges and opportunities for the application of scRNA-seq in plants.

## Introduction

Plants are multicellular eukaryotic organisms composed of various tissues, organs, and cell types that integrate and coordinate specific functions [1]. Traditional bulk RNA sequencing (RNA-seq) technologies are not sufficient for understanding cell-type-specific molecular mechanisms underlying the complexity of plants [2,3]. To address this limitation, single-cell RNA-seq (scRNA-seq) has been developed as a progressive technology for unraveling the transcriptomic variations that exist within cellular populations as well as capturing the dynamic changes in transcript abundance at the cellular level [4]. This new technology has multiple advantages over bulk RNA-seq: (a) scRNA-seq enables the identification and characterization of distinct cell types and states within a complex tissue or population of cells, providing a detailed understanding of cellular diversity that may be unobserved in bulk RNA-seq [5,6]; (b) scRNA-seq can identify rare cell types or subpopulations that might be imperceptible in bulk RNA-seq, allowing researchers to study their unique gene expression profiles and functional properties [7,8]; (c) scRNA-seq can capture the temporal changes in gene expression within individual cells, enabling the study of developmental processes, cellular responses to stimuli, or disease progression with higher resolution compared to bulk RNA-seq [9–11]; (d) scRNA-seq provides a relatively unbiased approach to study gene expression at the single-cell level, allowing for the detection of rare or unexpected gene expression patterns that might be missed in bulk RNA-seq, which relies on population-level averaging [12].

scRNA-seq has recently been used in plant systems biology research, allowing for the exploration of cellular dynamics and regulatory networks. For example, scRNA-seq has been applied in *Arabidopsis thaliana* to reveal root development patterns under a heat-shock condition [13], in *Zea mays* to gain new insights into cell fate determination during embryo development [14], in *Oryza sativa* and *Triticum aestivum* to understand gene expression in leaf and root cells under stress conditions [7,8,15], and in *Populus trichocarpa* to gain a deep understanding of the molecular mechanism underlying xylem differentiation in woody plants [16]. scRNA-seq is also emerging as a powerful technology to facilitate plant synthetic biology research in various aspects: (a) identifying cell-type-specific biological parts (e.g., promoters), and (b) deciphering plant regeneration mechanisms for overcoming the bottleneck of plant transformation. For instance, cell-type-specific promoters identified from scRNA-seq data can be used for cell-type-specific genome engineering, which will minimize negative pleiotropic effects [17].

Technical advancements in scRNA-seq in plants have been occurring rapidly, including the development of improved protocols for cell isolation, library preparation, and sequencing technologies [11,18]. Newer methods have been developed to handle plant-specific challenges such as rigid cell walls [11]. Moreover, computational tools and algorithms, including explainable-artificial intelligence (AI) approaches, have been refined to handle the large and complex datasets generated by scRNA-seq [19]. However, challenges persist, including the limited availability of comprehensive plant cell atlases, difficulties in capturing rare cell types, and the potential for technical noise due to low RNA contents [9–11].

There have been multiple excellent reviews summarizing the application of scRNA-seq in plants [11,20,21]. In this review, we provide an update on the technological development of scRNA-seq and an overview of recent advances in the application of scRNA-seq in plant systems and synthetic biology. Also, we discuss the opportunities and challenges associated with this exciting field to inspire further innovations in this rapidly evolving field, along with perspectives on how to accelerate the application of scRNA-seq in plant systems and synthetic biology research.

## Methods of Single-Cell Transcriptomics

**Fig. 1.**
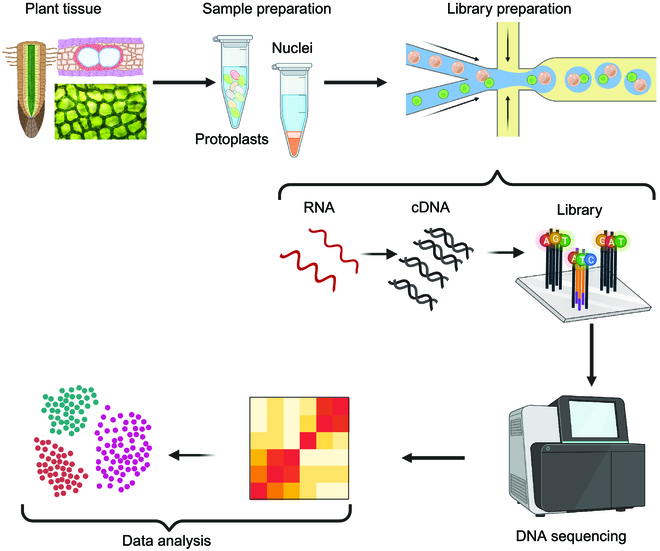
Conceptual workflow of single-cell RNA-sequencing (scRNA-seq). Starting from whole plant tissues and dissociating cells (protoplasts) or nuclei from the tissue, RNA extraction, cDNA synthesis by reverse transcription, library preparation followed by sequencing, expression abundance estimation, and cell-type identification. Redrawn from [151]. Created with BioRender.com.

scRNA-seq technology can be divided into 2 components: the experimental components involved in generating scRNA-seq data and the computational components related to the analysis of scRNA-seq data. A general workflow for scRNA-seq is illustrated in Fig. 1. In this section we provide an update on the experimental components, including sample preparation, scRNA-seq library construction, and sequencing. Then, we summarize the technical advancements in computational analysis of scRNA-seq data, along with scRNA-seq databases established for plants.

### Sample preparation for scRNA-seq

In plants, scRNA-seq experiments have been conducted using protoplasts and nuclei derived from roots, leaves, shoot apical meristems, stems, and inflorescences of various species. scRNA-seq using nuclei samples is also called single-nucleus RNA sequencing (snRNA-seq). Nevertheless, most studies have primarily employed protoplasts, as opposed to nuclei (Table 1). Protoplasts, by excluding the cell walls, allow for the capture of both nuclear and cytoplasmic RNAs. This comprehensive capture of the full transcriptome provides a more holistic view of gene expression patterns and regulatory processes within individual cells. However, protoplast preparation for scRNA-seq suffers from one major limitation: Enzymatic digestion of the cell wall poses a cellular stress that impacts gene expression. On the other hand, the isolation methods for nuclei selectively capture intact nuclei, facilitating a focused analysis of nuclear-encoded RNA transcripts in plants [22]. Compared to protoplast-based scRNA-seq methods, nuclei isolation can theoretically isolate complex cell types including those that are sensitive to cell degradation enzymes, thus potentially resulting in higher cell-type diversity [23]. Nevertheless, using nuclei samples for scRNA-seq may potentially miss valuable information from cytoplasmic RNAs. This limitation restricts the ability to investigate post-transcriptional modifications, cytoplasmic signaling, and other processes occurring outside the nucleus, which can be crucial for comprehensively understanding cellular functions.

**Table 1.** Summary of experimental methods for scRNA-seq in plants. The information about sample preparation, library construction, and sequencing platforms was obtained from a partial list of recent publications reporting scRNA-seq in plants.

Species	Sample preparation	Library construction	Sequencing platform	Reference
*Arabidopsis thaliana*	Root protoplast	Chromium Single Cell 3′ Reagent Kit v2 (10x Genomics)	Illumina NextSeq	[25]
*A. thaliana*	Root protoplast	Chromium Single Cell 3′ Reagent Kit v2 (10x Genomics)	Illumina HiSeq	[26]
*A. thaliana*	Root protoplast	Nextera XT DNA Library Kit (Illumina)	Illumina HiSeq and NextSeq	[32]
*A. thaliana*	Leaf protoplast	Chromium Single Cell 3′ Reagent Kit v2 and v3 (10x Genomics)	Illumina HiSeq	[30]
*A. thaliana*	Shoot protoplast	Chromium Single Cell 3′ Reagent Kits v3 (10x Genomics)	Illumina NovaSeq	[7]
*Oryza sativa*	Root protoplast	Chromium Single Cell 3′ Reagent Kit v3 (10x Genomics)	Illumina NovaSeq	[155]
*O. sativa*	Leaf protoplast	Chromium Single Cell 3′ Reagent Kit v2 (10x Genomics)	Illumina NovaSeq	[31]
*O. sativa*	Leaf and root protoplast	Chromium Single Cell 3′ Reagent Kit v3 (10x Genomics)	Illumina HiSeq	[15]
*O. sativa*	Inflorescence and leaf protoplast	The BD Rhapsody system	Illumina sequencer	[*34*]
*Zea mays*	Root protoplast	Chromium Single Cell 3′ Reagent Kit v3 (10x Genomics)	Illumina HiSeq	[156]
*Solanum lycopersicum*	Shoot apex protoplast	Chromium Single Cell 3′ Reagent Kit v3 (10x Genomics)	Illumina Novaseq	[137]
*Populus* spp.	Shoot apex and stem nuclei	Chromium Single Cell 3′ Reagent Kit v3.1 (10x Genomics)	Illumina NovaSeq	[22]

The utilization of microfluidics platforms is the primary workflow for single-cell separation, isolation, and analysis [24]. To ensure optimal performance, it is crucial that the protoplast or nuclei suspensions consist of well-dissociated, intact protoplasts/nuclei, devoid of any cell debris. Currently, the development of protoplast isolation methods compatible with microfluidics has primarily focused on *A. thaliana* [7,25,26]. As mentioned above, a major challenge in protoplast isolation is dissociating the cells from their rigid cell walls. Some tissues (e.g., xylem) in many species are recalcitrant to digestion [27,28]. Alternatively, nuclei can be isolated and purified using fluorescence-activated cell sorting (FACS) to address the challenges posed by enzymatic digestion of cell walls. FACS also offers advantages such as the utilization of frozen samples and reduced suspension preparation time [29]. Among the scRNA-seq platforms used in plants, both protoplasts and nuclei can be readily accommodated by 10x Genomics protocols compatible with different tissue types [15,22,25,26]. The choice between protoplasts and nuclei for plant scRNA-seq depends on the specific research goals, the plant species, and the trade-offs between transcriptome coverage, spatial information, and technical feasibility. Researchers should carefully consider these factors to determine the most suitable sample preparation method for their study.

### scRNA-seq library construction and sequencing

Various library preparation methods have been applied in plant scRNA-seq research, with the 10x Genomics system being widely used for constructing scRNA-seq libraries in diverse plants species (Table 1), which has been used to profile 5,000 to 20,000 cells from various plant tissues [25,26,30,31]. In addition to the commonly used 10x Genomics system, other library construction methods show great potential for scRNA-seq in plants. For example, the Nextera XT DNA Library Preparation Kit (Illumina) was used for scRNA-seq library construction to profile >12,000 cells from *A. thaliana* roots [32]. Recently, the BD Rhapsody system (BD Life Sciences, San Jose, USA), which utilizes microwell-based cartridges to capture a broad range of single cells, enabling simultaneous measurement of multiple aspects, including gene expression and protein abundance [33], was used to profile 37,571 cells from *O. sativa* inflorescence, resulting in a comprehensive gene expression atlas of early inflorescence development [34]. The scRNA-seq libraries constructed using various methods have been sequenced using the Illumina sequencing technologies, such as HiSeq, NextSeq, and NovaSeq (Table 1).

### scRNA-seq data analysis

**Fig. 2.**
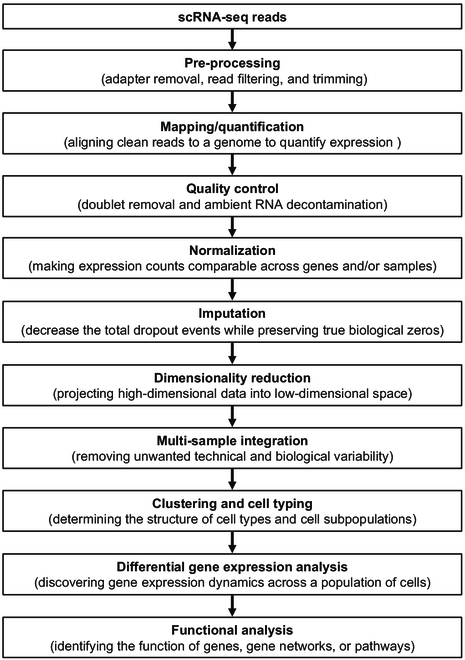
Diagrammatic representation of a scRNA-seq data analysis pipeline. Redrawn from [35–37].

scRNA-seq experiments generate complex, high-dimensional data that require specialized pipelines for analysis and interpretation [35]. scRNA-seq data analysis involves multiple steps, including pre-processing, alignment (or mapping), quality control, normalization, imputation, dimensionality reduction, integration, clustering, differential gene expression, and functional analysis [36–37], as illustrated in Fig. 2.

Pre-processing: The pre-processing step involves filtering out low-quality reads (e.g., reads having low quality bases in their unique molecular identifier [UMI]) and trimming low-quality bases [38,39].

Read alignment: The most common read mapping tools for scRNA-seq data are Cellranger [40], STARsolo [37,41,42], and Kallisto [43]. Cellranger contains a modified version of STAR that is equivalent but less efficient than STARSolo. However, there are numerous other choices for read alignment. Regardless of the choice of aligner, reads are aligned to a reference genome and collapsed by UMI to yield a gene-by-cell matrix of integer feature counts.

Quality control: The quality control step involves doublet/multiplet (libraries generated from 2 or more cells) removal and ambient RNA (mRNA derived from non-barcoded lysed cells) decontamination [35]. Doublets and multiplets severely confound scRNA-seq data analysis and several tools have been developed for their detection, such as DoubletFinder [44] and SoCube [37]. Ambient RNA contamination is an important issue for scRNA-seq, in particular with the approaches based on single-nucleus isolation that causes cell lysis [45]. Typically, a vast number of aligned cells are first omitted through several thresholding steps: a lower UMI cutoff (<500 UMIs), a low unique gene cutoff (<50 genes), and a low mitochondrial content cutoff (<5% to 10% or so, depending on the plant species and not applying to snRNA-seq).

Normalization: Normalization for scRNA-seq data typically happens in 2 steps. First, a scaling step divides feature counts for each cell by the total counts for that cell to address gene-specific biases such as relative gene abundances and then scales all expression values by the same amount. This step is similar to the typical bulk RNA-seq scaling step (e.g., counts per million [CPM] or transcripts per million [TPM]) and can be replaced by one of those methods, although scRNA-seq protocols, which use UMIs, do not exhibit the gene length bias that bulk RNA-seq methods do [46] and need not account for it. Second, a transformation is applied to all feature counts (typically a log transformation) for variance stabilization [36, 47, 48]. Between-sample normalization can be performed using 2 types of approaches: (a) global scaling approaches, including the simple CPM transformation and other more robust scaling procedures, such as DESeq2’s normalization method [49] and PsiNorm [50], or (b) non-linear approaches, such as Linnorm [51], sctransform [52], and Dino [53].

Imputation: scRNA-seq data are highly sparse, and it is difficult to determine whether observed zeroes correspond to biological or technical (so-called “dropout events”) zeroes [54]. However, various methods have been developed to decrease the total dropout events within a count matrix while preserving true biological zeros. These imputation methods fall into 3 major categories. The first category models the sparsity using a probabilistic model to impute gene expression for only the dropout events. The second category is applied to the whole count matrix by smoothing the gene expression values in cells with the identification of similar cell profiles. The third category reconstructs the whole count matrix from a latent space representation of the cell profiles identified by a deep-learning or low-rank matrix method [55]. A benchmarking study performed comparisons of 18 different imputation methods and found that single-cell variational inference (scVI) [56], deep count autoencoder network (DCA) [57], and Markov affinity-based graph imputation of cells (MAGIC) ranked higher than all other methods [58]. Additionally, this benchmarking study showed that k nearest-neighbors (kNN)-smoothing [59] and scVI [56] obtained the highest overlap of differentially expressed genes (DEGs) between scRNA-seq and bulk RNA-seq, consistently outperforming differential expression analysis performed on non-imputed data [55]. Furthermore, MAGIC [58], SAUCIE [60], and SAVER-X [61] ranked highest in improving unsupervised clustering results compared to no imputation applied [55].

Dimensionality reduction: The dimensionality reduction step projects high-dimensional data into low-dimensional space for visualizing the structure of cell clusters and inferring cell developmental trajectories [62]. Various methods for dimension reduction have been developed using linear and non-linear model-based approaches and deep learning algorithms. The most commonly used method is Uniform Manifold Approximation and Projection, which results in superior organization for cell-type clusters and has the lowest computational cost [63]. Recently, some new methods have been developed for dimensionality reduction, such as DREAM, which captures the differences between cell types for automatically learning the hierarchical representation of input data and models dropout events [64], and PHATE, which generates lower-dimensional embeddings that are quantitatively better denoised than existing visualization methods [65].

Integration: Integration can either be performed across samples or across conditions. The multi-sample integration step removes unwanted technical and biological variability [35]. Various tools have been developed for integration of scRNA-seq data-derived heterogeneous samples, such as batch balanced k nearest neighbors (BBKNN) [66], Harmony [67], Seurat CCA [68], and Scanorama [69], among which Scanorama has the best performance [35].

Clustering and cell typing: In order to assign cells to a cell type or state within an scRNA-seq experiment, they are separated into clusters in which they share similar gene expression patterns. Clustering is composed of 2 steps, generating a graph from the gene-by-cell count matrix and then clustering that graph. A widely used graph embedding method is the kNN method [70], although other graph generation methods can be used, including explainable-AI methods such as iRF-LOOP and CoMet [71]. There are numerous commonly used graph clustering methods, among which k-means, hierarchical clustering [72], the Leiden algorithm [73], and Markov Clustering [74] are some of the most common ones. Once cells have been separated into clusters based on the underlying graph topology, each cluster is assigned a cell type based on the top DEGs within that cluster.

Differential expression and functional analysis: Differential expression analysis discovers the changes in gene expression across a population of cells, which exhibits multimodal and heterogeneous patterns resulting from different cell types, different mRNA contents, and different cell states [75]. A recent comprehensive benchmarking analysis [76] demonstrated that single-cell differential gene expression methods based on negative binomial mixed models, such as glmmTMB [77] and NEBULA-HL [78], outperformed several other methods. Then, functional analysis can be performed to infer the function of genes, gene modules/networks, or pathways at the cell-type level. In general, this step includes gene set enrichment analysis and functional enrichment analysis [37].

### scRNA-seq databases

The fast accumulation of scRNA-seq data for various plant species generates the needs for establishing online databases to facilitate the public sharing and utilization of scRNA-seq results. Over recent years, an increasing number of plant-specific scRNA-seq databases have been created, with some of them listed in Table 2. For example, a database, called PlantscRNAdb, was recently developed for analyzing scRNA-seq data in plants, which contains 26,326 marker genes of 128 different cell types from *A. thaliana*, *O. sativa*, *Solanum lycopersicum*, and *Z. mays* [79]. More recently, another database, called Plant Single Cell Transcriptome Hub (PsctH), was created to provide a comprehensive and accurate resource of cell markers and web tools for various cell types in plant tissues, which include 98 cell markers from 51 cell types in 9 plant tissues/sub-tissues of *A. thaliana*, *Arachis hypogaea*, *O. sativa*, *S. lycopersicum*, and *Z. mays* [80]. Another database, called Plant Cell Marker DataBase (PCMDB), which contains 81,117 cell marker genes of 263 cell types in 22 tissues across 6 plant species (*A. thaliana*, *Glycine max*, *Nicotiana tabacum*, *O. sativa*, *S. lycopersicum*, and *Z. mays*), was created through manual curation of experimentally validated marker genes, differentially expressed marker genes based on both bulk RNA-seq data and scRNA-seq data, facilitating the cell typing in scRNA-seq data analysis [81]. Most of the plant scRNA-seq databases contain cellular gene expression in a limited number (<7) of species. To address this limitation, He et al. [82] established a comprehensive database, called scPlantDB, which covers ~2.5 million cells across 17 plant species, facilitating the analysis and utilization of multiple scRNA-seq datasets.

**Table 2.** A partial list of scRNA-seq databases

Database name	Website	Species	Reference
PlantscRNAdb	http://ibi.zju.edu.cn/plantscrnadb/	*Arabidopsis thaliana*, *Oryza sativa*, *Solanum lycopersicum*, and *Zea mays*	[79]
3D Flower Meristem	http://threed-flower-meristem.herokuapp.com/	*A. thaliana*	[157]
The Plant scRNA-Seq Browser (PscB)	https://www.zmbp-resources.uni-tuebingen.de/timmermans/plant-single-cell-browser/	*A. thaliana*	[140]
Plant Single-Cell Transcriptome Hub (PsctH)	http://jinlab.hzau.edu.cn/PsctH/	*A. thaliana*, *Z. mays*, *O. sativa*, *Arachis hypogaea*, and *Solanum lycopersicum*	[80]
Root Cell Atlas	https://phytozome-next.jgi.doe.gov/tools/scrna/	*A. thaliana*	[6]
stRNAPal	https://pgx.zju.edu.cn/stRNAPal/	*Populus alba × P. glandulosa* clone 84K	[85]
PCMDB	https://www.tobaccodb.org/pcmdb/	*A. thaliana*, *Glycine max*, *Nicotiana tabacum*, *O. sativa*, *S. lycopersicum*, and *Z. mays*	[81]
scPlantDB	https://biobigdata.nju.edu.cn/scplantdb/	*A. thaliana*, *Bombax ceiba*, *Brassica rapa*, *Catharanthus roseus*, *Fragaria vesca*, *G. max*, *Gossypium bickii*, *G. hirsutum*, *Manihot esculenta*, *Medicago truncatula*, *N. attenuata*, *O. sativa*, *P. alba* var. pyramidalis, *P. alba* × *P. glandulosa*, *S. lycopersicum*, *Triticum aestivum*, and *Z. mays*	[6,82]

## Application of scRNA-seq in Plant Systems Biology

**Fig. 3.**
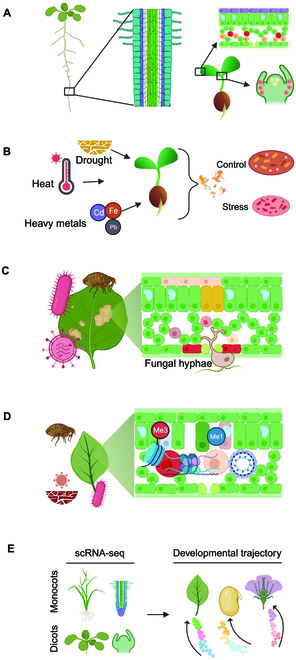
Some recent applications of single-cell RNA-seq in plant system biology. (A) A developmental gradient of cell types from root, leaf, and shoot apical meristem. Redrawn from [7,146]. (B) The effect of abiotic stress on cellular-level transcriptomics. Redrawn from [152]. (C) The effect of different biotic stresses on cell types within a complex organ. Redrawn from [8]. (D) Epigenetic regulation of different cell types under different environmental stimuli. Redrawn from [153]. Me1, mono-methylation; Me3, tri-methylation. (E) Cell fate determination of root and shoot apical meristem into different tissues. Redrawn from [154]. Created with BioRender.com.

Plant systems biology is an interdisciplinary field that uses computational and experimental approaches to study complex plant biological systems, with a focus on understanding how individual components of the system interact and contribute to the overall behavior of the system [83]. Profiling the transcriptomes of plant cells, tracking their dynamic changes at different plant cell development stages, and illuminating how they respond to environmental stresses can reveal developmental and stress-response mechanisms at the cellular level [21]. In this section, we highlight some recent applications of scRNA-seq in plant systems biology research, as illustrated in Fig. 3.

### Understanding the molecular mechanism of plant development at the cellular level

Dynamic cell identities contribute to the plant developmental process [30]. To understand plant development, it is necessary to reveal the full extent of cell types, developmental domains, and the regulatory networks that control their differentiation [84]. scRNA-seq is a powerful tool to investigate the cell-specific transcriptomics. To date, scRNA-seq experiments have been carried out to reveal detailed maps of the cell types in various types of plant tissues. For example, most of the major cell types of stem-differentiating xylem were identified using scRNA-seq in *P. trichocarpa* [16]. Also, a recent study integrating high-resolution anatomical analysis and spatial transcriptomics characterized the meristematic cell developmental trajectory from primary to secondary vascular tissues in *P. alba* × *P. glandulosa* clone 84K stems and revealed rectangular procambium-like cells in the phloem domain and fusiform cambium zone cells in the xylem domain [85]. In *A. thaliana*, cell clusters representing the below- and aboveground tissue were identified by scRNA-seq, providing new insights into the spatial control of gene expression at the cellular level at defined time points [86]. Furthermore, scRNA-seq can be used to investigate the role of transcription factors in plant development. For example, scRNA-seq was recently used to generate a cell-resolution developmental map of the maize root, demonstrating that a hypermobile transcription factor SHORT-ROOT (SHR), which regulates the expansion of cortical tissue, travels at least 8 cell layers into the cortex in maize [87]. Recently, scRNA-seq was employed to demonstrate that 2 transcription factors responsive to brassinosteroids, namely, HOMEOBOX FROM ARABIDOPSIS THALIANA 7 (HAT7) and GT-2-LIKE 1 (GTL1), are associated with cortex elongation [88].

### Understanding the plant responses to abiotic and biotic stresses at the cellular level

Plants are exposed to a variety of abiotic and biotic stresses including drought, flood, salinity, freezing/cold, heat, and different pathogens [89]. Specific cell types respond differently to environmental conditions, with some surviving more severe stresses than others, but it is still unclear how specific cell types respond to different environmental cues and communicate with other cells for improved survival [11]. scRNA-seq has been used to investigate how abiotic stress changes the cell-type-specific gene expressions in plants. For example, scRNA-seq analysis of *A. thaliana* seedlings under heat stress revealed that while heat response expression was dominated by canonical heat-shock genes across cell types, there were subtle but substantial differences in the expression of other genes among cell types [13]. Also, scRNA-seq analysis of both shoot and root of *O. sativa* seedlings exposed to various abiotic stresses (high salinity, low nitrogen, and iron deficiency) showed that abiotic stresses affected gene expression largely in a cell-type-specific manner whereas roughly the same set of genes were regulated at the transcriptional level by different stresses for a given cell type [15].

scRNA-seq has also been used to study plant responses to biotic stresses caused by bacterial and fungal pathogens as well as insects. For example, a recent study used scRNA-seq to profile the transcriptome of *A. thaliana* leaf tissue infected with the bacterial pathogen *Pseudomonas syringae* and revealed distinct pathogen-responsive cell clusters exhibiting transcriptional responses at immune, transition, and susceptible states, providing insights into molecular mechanism of disease progression [90]. Also, an scRNA-seq analysis of *A. thaliana* leaf tissue unveiled cell-type-specific gene expression in response to the fungal pathogen *Colletotrichum higginsianum*, providing insights into the role of the epidermis-expressed MYB122 in disease resistance [91]. Furthermore, a comparative scRNA-seq analysis of rice varieties in response to the brown planthopper (*Nilaparvata lugens*), which sucks rice sap causing leaves to turn yellow and wither, revealed significant differences in cell types (e.g., mestome sheath cells, guard cells, mesophyll cells, xylem cells, bulliform cells, and phloem cells) in the rice resistance to the brown planthopper between susceptible (TN1) and resistant (YHY15) rice varieties [92].

### Understanding epigenetic regulation in plants at the cellular level

Epigenetic modifications, such as DNA methylation and histone modifications, play important roles in regulating gene expression in plants. Although epigenetic regulation of global gene expression has been widely studied [93,94], the role of epigenetic regulation at the single-cell level has not been deeply explored in plants. Single-cell epigenomics and its emerging applications are being studied mainly in animals [95–97]. For example, scRNA-seq, along with the assay for transposase-accessible chromatin with sequencing (ATAC-seq), has been used to profile mouse cardiac progenitor cells, providing a deep understanding of transcriptional and epigenetic regulations during cardiac progenitor cell fate decisions [98]. Similarly, an integrative scRNA-seq and ATAC-seq analysis of human immunophenotypic blood cells revealed that extensive epigenetic but not transcriptional priming occurred in hematopoietic stem cells/multipotent progenitors prior to the commitment of their lineages [99]. Inspired by the examples from mammalian research, the same strategies can be applied in single-cell epigenomics in plants. scRNA-seq can be combined with epigenetic profiling techniques, such as chromatin immunoprecipitation sequencing (ChIP-seq), ATAC-seq, or bisulfite sequencing, to analyze the relationship between gene expression and epigenetic modifications at the single-cell level [100]. For instance, an integrative snRNA-seq and ATAC-seq analysis of wheat root samples revealed asymmetric gene transcription and a cell-type-specific gene regulatory network driving root hair differentiation [101]. Overall, scRNA-seq is emerging as a powerful tool for providing insights into the relationship between gene expression and epigenetic regulation in plants.

### Understanding cell fate determination and organogenesis in plants

Plant cell fate determination is a complex process that involves various signaling pathways and regulatory mechanisms. During growth, differentiation of a cell into a specific cell type is determined by the expression of specific genes, which are controlled by various transcription factors and signaling molecules [102]. scRNA-seq has great potential for unraveling the molecular mechanisms underlying cell fate determination in plants. For example, a recent study used scRNA-seq to profile gene expression in the ovule outer integument of *Gossypium hirsutum* at 4 developmental stages and identified 2 transcription factors (MYB25-like and HOX3) as key players in fiber differentiation and tip-biased diffuse growth, respectively [103]. Also, a recent scRNA-seq analysis of hypocotyl tissue in regenerable cotton genotypes Jin668 and recalcitrant TM-1 revealed the significant role of the primary vascular cell type in undergoing cell fate transitions in response to external stimuli and identified novel regeneration-associated genes, such as CSEF, PIS1, AFB2, ATHB2, PLC2, and PLT3 [104]. To investigate the cell development in xylem, which is the predominant plant tissue facilitating lateral growth, Tung et al. [105] performed a comparative spatial transcriptomic analysis of 4 plant species, including 2 core eudicots (*P. trichocarpa* and *Eucalyptus grandis*), one basal eudicot (*Trochodendron aralioides*), and one basal angiosperm (*Liriodendron chinense*), using scRNA-seq in combination with laser capture microdissection transcriptomics, providing a deep understanding of the formation of xylem cell lineages in diverse plant species across a large evolutionary space.

## Application of Single-Cell Genomics in Plant Synthetic Biology

**Fig. 4.**
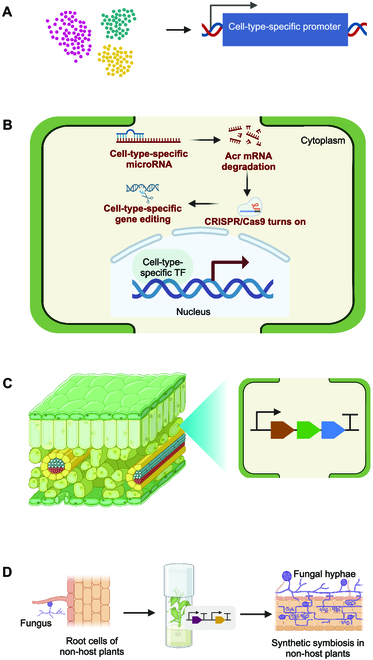
A conceptual framework for the application of single-cell RNA-seq in plant synthetic biology. (A) Discovering and designing promoters for cell-type-specific gene expression. (B) Discovering regulators (e.g., miRNA and TF) for enabling cell-type-specific CRISPR/Cas-based gene-editing or cell-type-specific gene expression. Acr, anti-CRISPR protein; TF, transcription factor; CRISPR/Cas9, clustered regularly interspaced short palindromic repeats/CRISPR-associated protein 9. (C) Cell-type-specific metabolic pathway engineering in plants. (D) Engineering of synthetic symbiosis between non-host plants and microbes. Created with BioRender.com.

Plant synthetic biology is a rapidly growing field that contributes to the design and creation of new plant traits [17,106]. With its potential to revolutionize agriculture, improve ecosystem sustainability, and advance overall plant health, plant synthetic biology is an exciting area of research that promises to benefit society in numerous ways [107]. By comprehending the intricate cellular dynamics of plant biosystems, it becomes possible to execute precise biodesign at the cellular level. In this section, we highlight the applications of scRNA-seq for discovering new cell-specific molecular components for cell-specific plant biodesign, revealing biosynthesis pathways for metabolic engineering, and engineering novel symbiosis between plants and microbes, as illustrated in Fig. 4.

### Discovering and designing cell-type-specific promoters for plant bioengineering

From an engineering perspective, cell-type-specific promoters can be defined as promoters that could reproducibly drive high levels of transgene expression in a particular cell type [108]. Plants are multicellular organisms that contain many specialized types of cells, with each cell type having a specific function and position within an organ [109]. In plant bioengineering, cell-type-specific promoters have substantial advantage over constitutive ones (e.g., CaMV35S), which can generate unintended impacts on plants due to pleiotropic effects [110,111]. To minimize adverse pleiotropic effects in plant biodesign, cell-type-specific promoters are needed for precise spatial control of gene expression at the cell-type level. Cell-type-specific promoters have been widely applied for plant genetic engineering [112,113]. Specifically, cell-type-specific promoters can be designed based on the DNA sequences (e.g., cis-elements) upstream the genes showing cell-type-specific gene expression in plants, as revealed through scRNA-seq analysis. These promoters can be used to enable cell-type-specific gene regulation (e.g., overexpression and downregulation) or gene editing to engineer targeted traits (e.g., cell-typic specific metabolism and cell-typic development) while avoiding or minimizing the influence on non-targeted traits. For example, cell-type-specific gene editing has been achieved using cell-type-specific promoters to drive the expression of the CRISPR/Cas9 system [114,115]. To minimize the growth defects caused by overexpression of lignin biosynthesis repression gene, a xylem-fiber-specific promoter was used to express the repressor specifically in fiber cells [116]. Hence, discovering and designing additional cell-type-specific promoters will aid in the advancement of plant biodesign.

The identification of cell-type-specific promoters often requires gene expression profiling at the cellular level [117]. scRNA-seq allows for the identification of cell-type-specific gene expression, providing genomic information for cloning or redesigning cell-type-specific promoters [14]. For example, 2 vessel and fiber-specific genes, *PtWAT1* (POPTR-002G029100) and *PtCesA8* (POPTR-004G059600), were discovered from scRNA-seq profiles of stem-differentiating xylem in *P. trichocarpa*, which were validated by characterizing the expression of a β-glucuronidase (GUS) reporter under the control of these promoters in transgenic *P. tomentosa* plants [16]. Recently, many additional new cell types and cell identity marker genes have been identified via scRNA-seq analysis in various plant species [118,119], facilitating the design of new cell-type-specific promoters. Finally, together with chromatin profiling methods (e.g., ATAC-seq), scRNA-seq enables the discovery of cis-regulatory elements (CREs) within the genome [14,120]. For example, a large number of CREs have been discovered from root cells in *A. thaliana* and different organs of maize based on scRNA-seq data [14,121]. Those cell-type-specific CREs provide valuable components for building synthetic cell-type-specific promoters for plant biodesign.

MicroRNAs (miRNAs), another type of critical regulators of gene expression, are involved in numerous biological processes in both animals and plants [122]. Recently, a cell-specific Cas-ON switch based on miRNA-regulated expression of anti-CRISPR (Acr) proteins was developed in human cells, in which cell-specific target sites for miRNAs were inserted at the 3′ UTR (untranslated region) of Acrs and thus resulted in Acr knockdown to release Cas9 activity in specific cells [123]. Hence, miRNA-regulated cell-type-specific expression offers a great potential for cell-specific genome editing. With demonstrated activity of Acrs for inhibiting the CRISPR/Cas9 system in various plant species [124], a cell-type-specific Cas-ON switch can also be built using the same strategy as the Cas-ON switch in human cells. scRNA-seq has previously been used to profile the miRNAs in human cells, fly, and nematode [125,126], and thus, it may be possible to use this approach to identify cell-type-specific miRNAs in plants.

### Designing strategies for plant natural product engineering

Plant systems are becoming an important chassis platform for the high-yield production of complex plant natural products (PNPs) [127]. Identification of the key enzymes in biosynthetic pathways is essential for the heterologous production of PNPs [127]. scRNA-seq allows for exploring the multicellular compartmentation of specialized metabolisms in plants by profiling the transcriptomics at the cellular level [128]. Recently, a scRNA-seq analysis of the biosynthetic pathway for monoterpenoid indole alkaloids (MIA) in *Catharanthus roseus* revealed that MIA biosynthesis started in internal phloem-associated parenchyma cells, with the following enzymatic steps of the pathway preferentially occurring in epidermal cells and the last steps occurring in idioblast cells [128]. In addition, the intermediate transporters between distinct cell types were also detected [128]. Another recent example of the application of scRNA-seq in PNP engineering is the creation of single-cell transcriptome atlas in *Taxus mairei* stems, which, in combination with mass spectrometry imaging, identified multiple genes controlling the cell-specific gene expression involved in the biosynthesis of taxol, providing new knowledge for engineering of taxol production [129]. Thus, scRNA-seq has great potential for elucidating the spatial distribution of PNPs biosynthesis, transportation, and storage, providing detailed guidance for fine-tuning metabolic pathways in plants to enhance PNP production.

### Designing and engineering of nitrogen-fixing nodule symbiosis

Nitrogen-fixing soil bacteria, called rhizobia, can provide a steady supply of nitrogen for legumes via symbiosis [130]. This symbiosis, also called nodulation, begins with the infection of root hair cells by the bacteria, which triggers the formation of nodule primordia from root cortical, endodermal, and pericycle cells, prompting the formation of a new root organ, the nodule [130]. Since the discovery of this nodule endosymbiosis with nitrogen-fixing rhizobia, there has been a broad interest in transferring this trait to non-leguminous crops [131]. The most feasible approach to engineering a nitrogen-fixing root nodule symbiosis would likely involve emulating an established symbiotic relationship through gene transfer from nodulating to non-nodulating species [131]. Plant synthetic biology has a great potential for engineering nitrogen-fixing nodule symbiosis through coordinated genetic programming of multiple aspects, including Nod factors recognition, bacterial infection, root nodule formation, and creation of an appropriate atmosphere for nitrogenase activity within the nodule [132].

Inspired by various nitrogen-fixing symbioses, the engineering of nitrogen-fixing nodule symbiosis encompasses a diverse range of biochemical pathways and cell types [131]. To date, several core sets of symbiosis genes have been identified via omics and functional genomics studies [133,134]. However, due to the limited resolution of transcriptomic analyses at the whole tissue level, the accurate picture of the development of nitrogen-fixing nodule symbiosis remains unclear. scRNA-seq has a great potential for capturing the transcriptomic programs controlling the nodulation process at the cellular level. Recently, a scRNA-seq analysis of the *Medicago truncatula* root at an early stage after inoculation established a high-resolution spatial transcriptomic map, leading to the discovery of many novel genes and functional pathways controlling the response of *Medicago* root to rhizobium inoculation [130].

## Challenges and Potential Solutions for the Application of scRNA-seq in Plants

Although scRNA-seq holds huge promise for plant systems and synthetic biology research, there are technical challenges in sample preparation, library construction, data analysis, and data sharing/utilization. Plant cells are surrounded by rigid cell wall, which can interfere with the isolation of intact nuclei or protoplasts for scRNA-seq. Efficient cell dissociation methods still need to be further developed for the preparation of nuclei or protoplast samples [135]. The yield and viability of isolated protoplasts can be influenced by various factors, such as tissue quality, genotype, physiological state, and stress response. One solution to this challenge is to optimize the isolation conditions, including the enzyme treatment duration, temperature, and osmotic potential, for improving protoplast yield and viability [136].

snRNA-seq has advantages over scRNA-seq for profiling complex plant tissues, especially when plant cell walls pose challenges for protoplast isolation [137]. Recent studies have reported protocols for isolating high-quality nuclei compatible with high-throughput snRNA-seq [137]. However, isolation of intact plant nuclei can be challenging due to their small size and susceptibility to damage. Maintaining the integrity of the nuclei during the isolation process requires careful handling and optimization of the lysis buffer composition and centrifugation parameters [138]. One technical challenge with the widely used droplet-based methods for scRNA-seq library construction, such as the 10x Chromium method, is that in a few cases, there are multiple cells or nuclei inside a droplet or a suboptimal number of beads inside a droplet, and therefore future technical improvement will be needed to ensure that one droplet contains one bead and one cell or nucleus [139].

Plant cells, being larger in size, pose a potential challenge in scRNA-seq studies, and no ideal cell preparation methods have been reported thus far [140]. A potential solution to this challenge is to apply some new scRNA-seq technologies (developed for animal systems) in plants. For example, a recent breakthrough in scRNA-seq methods called RevGel-seq has been reported, eliminating the need for specific single-cell RNA instruments during sample preparation in mammalian cells [141]. Traditional sample preparation for scRNA-seq relies on dedicated and instrument-dependent methods, but RevGel-seq, based on cell-barcoded bead complexes, has demonstrated convenient and efficient sample preparation for scRNA-seq in human and mouse cells without requiring any specific instrument. Comparing this method to the widely used 10x Chromium method and employing the same data analysis platform, researchers identified the same cell types with a consistent hierarchy of abundance. These findings suggest that RevGel-seq could serve as a viable alternative to the 10x Chromium method, although it has not yet been tested in plant cells. The RevGel-seq also offers a promising advantage by allowing for an early stopping point during sample preparation, enabling flexibility in sample collection, and processing at different times or locations. There are other, cheaper technologies developed in animal systems that could be applied to plants, such as split-pool ligation-based transcriptome sequencing (SPLiT-seq), which profiles single-cell transcriptomes via combinatorial barcoding with no need for the physical isolation of each cell [142,143], and particle-templated instant partition sequencing, which has no requirement for microfluidic devices [144]. Furthermore, a new method, called “vast transcriptome analysis of single cells by dA-tailing” (VASA-seq), was recently developed to capture data across the full length of both non-polyadenylated and polyadenylated transcripts in mice [145]. Compared to transcript 3′-end sequencing, VASA-seq has a considerable potential for application in plant single-cell samples to provide more comprehensive transcript information.

The lack of well-characterized and cell-type-specific marker genes in non-model species is a major challenge for scRNA-seq data analysis [143]. New progress is being made to solve this challenge, as demonstrated by the development of the PCMDB, which contains 81,117 cell marker genes of 263 cell types in 22 tissues across 6 plant species [81]. Another challenge for scRNA-seq data analysis is that many plant species have incomplete or poorly annotated reference genomes, which can hinder the accurate mapping and interpretation of scRNA-seq data. Cell-to-cell heterogeneity necessitates the generation of large datasets and robust bioinformatics pipelines for accurately identifying distinct cell types [146]. However, currently there is a lack of computational methods for classifying cell-type homologies and diversifications using scRNA-seq data derived from a large number of plant species [136]. One solution to this challenge is to establish a standardized plant spatial single-cell genomics database, incorporating up-to-date T2T (Telomere-to-Telomere) genomes, gene annotations, and scRNA-seq data from multiple species across various developmental phases and environmental conditions in a standardized format [147].

## Conclusion and Perspectives

scRNA-seq has emerged as a groundbreaking technology with broad applications in plant systems biology and synthetic biology. Preparation of high-quality protoplasts or nuclei samples is critical for the success of scRNA-seq. The choice between protoplasts and nuclei for scRNA-seq analysis in plants is influenced by research goals, plant species, and the balance of transcriptome coverage, spatial information, and technical feasibility. Protoplasts provide a comprehensive view of the transcriptome, while nuclei isolation methods focus on nuclear-encoded RNA transcripts. However, protoplast and nuclei isolation from woody plants are very challenging due to cell wall digestion. Future efforts should prioritize the development of optimized protoplast isolation methods, particularly for woody plants.

**Fig. 5.**
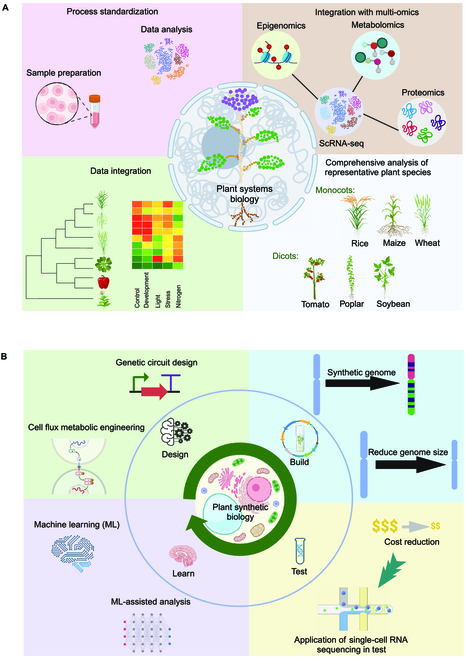
Perspectives on the application of single-cell RNA-seq (scRNA-seq) in plant systems biology and synthetic biology. (A) Opportunities for the application of scRNA-seq in plant systems biology, including standardization of scRNA-seq processes, integration of scRNA-seq with other omics technologies, application of scRNA-seq in various plant species, and data integration. (B) Opportunities for the application of scRNA-seq in the Design–Build–Test–Learn (DBTL) cycles of plant synthetic biology, including designing genetic circuits, building synthetic genomes, testing engineered systems, and learning new knowledge for the next iteration of DBTL. Created with BioRender.com.

There are a lot of opportunities for advancing the future application of scRNA-seq in plant systems biology research, as illustrated in Fig. 5A. With the application of scRNA-seq being rapidly expanded in plant systems biology and synthetic biology research worldwide, there is an urgent need for the standardization of scRNA-seq processes, including sample preparation, library construction, sequencing, and data analysis, across different laboratories and plant species. Efforts have begun on the generation of standard scRNA-seq process [148]. Developing standardized protocols and quality control measures will ensure reproducibility and comparability of scRNA-seq data, enabling more robust analyses and reliable conclusions.

Another promising direction is the integration of scRNA-seq with other omics technologies, as demonstrated in a recent report on the integration of scRNA-seq and ChIP-seq/ATAC-seq to build transcriptional networks in *Z. mays* [84]. Multiple computational tools have been developed to integrative analysis of heterogeneous single-cell multi-omics data, such as GLUE (graph-linked unified embedding) [149] and Con-AAE (Contrastive cycle adversarial Autoencoders) [150]. The multi-omics data integration strategy will enhance the comprehensive understanding of plant cellular processes and regulatory networks, allowing researchers to unravel complex interactions and mechanisms governing plant biology at a systems level.

The application of scRNA-seq needs to be expanded to a broader range of plant species beyond well-studied model organisms. This will not only reveal the biological processes in specific plant species but also benefit the discovery of the conserved regulatory networks across the plant kingdom. Data integration and analysis methods need to be further developed to handle the vast amount of scRNA-seq data generated from diverse plant species and experimental conditions.

In the future, scRNA-seq will play a significant role in advancing plant synthetic biology by generating high-resolution gene expression data to enhance the Design–Build–Test–Learn (DBTL) capability for plant synthetic biology, as illustrated in Fig. 5B. It will provide unprecedented new insights into the biological processes and gene expression regulation at the cellular level, which can be used to improve the efficiency, predictability, and stability of genetic engineering in plants. Researchers can unlock new possibilities for synthetic biology applications by utilizing scRNA-seq data for designing synthetic genomes or reducing genome size. Machine learning-assisted analysis of scRNA-seq data can be leveraged to gain insights into the functional roles of individual genes and their interactions, enabling the design of more precise and efficient synthetic genomes tailored to specific applications. So far, scRNA-seq has not been widely used for testing engineered plants due to the high cost of this new technology. It can be expected that new technical advancements will significantly reduce the cost of scRNA-seq analysis in the years to come, and consequently allow more and more researchers to study gene expression in genetically modified plants at the cellular level.
